# Urinary tract infection after radiation therapy or radical prostatectomy on the prognosis of patients with prostate cancer: a population-based study

**DOI:** 10.1186/s12885-023-10869-4

**Published:** 2023-05-03

**Authors:** Jihye Hyun, Moon Soo Ha, Seung Young Oh, Jong Hyun Tae, Byung Hoon Chi, In Ho Chang, Tae-Hyoung Kim, Soon Chul Myung, Tuan Thanh Nguyen, Jung Hoon Kim, Jin Wook Kim, Yong Seong Lee, Jooyoung Lee, Se Young Choi

**Affiliations:** 1 Department of Applied Statistics, Chung-Ang University, 84 Heukseok-Ro, Dongjak-Gu, 06974 Seoul, Republic of Korea; 2 Department of Urology, Hyundae General Hospital, Chung-Ang University College of Medicine, 21 Bonghyeon-ro, Gyeonggi-Do 12013 Namyangju-si, Republic of Korea; 3 Department of Urology, Chung-Ang University Hospital, Chung-Ang University College of Medicine, 102, Heukseok-Ro, Dongjak-Gu, 06973 Seoul, Republic of Korea; 4 Department of Urology, Cho Ray Hospital, University of Medicine and Pharmacy, Ho Chi Minh City, Vietnam; 5 Department of Urology, Chung-Ang University Gwangmyeong Hospital, Chung-Ang University College of Medicine, 110, Deokan-ro, Gyeonggi-Do 14353 Gwangmyeong-si, Republic of Korea

**Keywords:** Urinary tract infection, Mortality, Risk factor, Prognosis, Radiation therapy, Radical prostatectomy

## Abstract

**Background:**

We aimed to assess the trends in urinary tract infections (UTIs) and prognosis of patients with prostate cancer after radical prostatectomy (RP) and radiation therapy (RT) as definitive treatment options.

**Methods:**

The data of patients diagnosed with prostate cancer between 2007 and 2016 were collected from the National Health Insurance Service database. The incidence of UTIs was evaluated in patients treated with RT, open/laparoscopic RP, and robot-assisted RP. The proportional hazard assumption test was performed using the scaled Schoenfeld residuals based on a multivariable Cox proportional hazard model. Kaplan–Meier analysis were performed to assess survival.

**Results:**

A total of 28,887 patients were treated with definitive treatment. In the acute phase (< 3 months), UTIs were more frequent in RP than in RT; in the chronic phase (> 12 months), UTIs were more frequent in RT than in RP. In the early follow-up period, the risk of UTIs was higher in the open/laparoscopic RP group (aHR, 1.63; 95% CI, 1.44–1.83; *p* < 0.001) and the robot-assisted RP group (aHR, 1.26; 95% CI, 1.11–1.43; *p* < 0.001), compared to the RT group. The robot-assisted RP group had a lower risk of UTIs than the open/laparoscopic RP group in the early (aHR, 0.77; 95% CI, 0.77–0.78; *p* < 0.001) and late (aHR, 0.90; 95% CI, 0.89–0.91; *p* < 0.001) follow-up periods. In patients with UTI, Charlson Comorbidity Index score, primary treatment, age at UTI diagnosis, type of UTI, hospitalization, and sepsis from UTI were risk factors for overall survival.

**Conclusions:**

In patients treated with RP or RT, the incidence of UTIs was higher than that in the general population. RP posed a higher risk of UTIs than RT did in early follow-up period. Robot-assisted RP had a lower risk of UTIs than open/laparoscopic RP group in total period. UTI characteristics might be related to poor prognosis.

**Supplementary Information:**

The online version contains supplementary material available at 10.1186/s12885-023-10869-4.

## Introduction

Prostate cancer (PC) is known as the most common cancer in males and the second-most common cause of deaths in the United States [1]. In PC without distant metastasis, representative curative treatments include radical prostatectomy (RP) and radiation therapy (RT) with or without androgen deprivation therapy (ADT) as the definitive therapy. RP and RT exhibited survival gains over noncurative treatment in randomized controlled trials (RCTs) [2, 3]. In many observational studies, survival is better in RP than in RT [4]. Because RT-treated patients usually have worse tumor and patient characteristics than RP-treated patients, possibly affecting survival results [5]. In a recent RCT comparing active surveillance, RP, and RT, although patients have low- or favorable intermediate-risk localized PC, their cancer-specific survival at a median of 10-year follow-up does not differ [6]. Therefore, definitive treatment can be chosen on the basis of adverse effects or accessibility instead of cancer-specific survival.

Urinary tract infection (UTI) is one of the most common infections. One-fifth of all UTIs have been reported to occur in males, and the incidence of reported UTIs is 0.05 per person-year in males aged 65–74 years [7]. Urosepsis-related mortality increases to 20–40% according to age [8]. UTIs account for the largest proportion of nosocomial infections (approximately 36%) [9] that can affect mortality and social and medical costs [10]. In prostate biopsy, many strategies (e.g., perineal biopsy, target biopsy, and use of rectal swabs) have been applied to reduce infective complications [11]. However, in definitive PC treatment, infectious complications have been rarely considered. The incidence and risk factors of UTIs after treatment remain unclear. UTIs in prostate cancer can have several potential causes which are catheterization, urinary tract obstruction, hormone therapy, or reduced immunity by treatments [12]. Hence, we assessed the trends in UTIs and prognosis of patients with PC treated with RP and RT as definitive treatment options.

## Materials and methods

### Database

This study used the national health claims database released by the National Health Insurance Service (NHIS) that offers comprehensive medical care coverage to 99% of Koreans (over 50 million individuals). The database consists of information on record about inpatient and outpatient diagnosis and prescription by NHIS as single public insurer. Disease codes are identified by the Korean Standard Classification of Diseases and Causes of Death, 8th edition (KCD-8). For the estimated incidence in the general population, we used the Health Insurance Review and Assessment Service National Patient Sample (HIRA-NPS), which is a stratified random sample of 3% of the Korean population and contains information about patients’ diagnosis, treatment details, procedures, surgical history, and prescribed medications [13]. We used data from the HIRA-NPS of 2018, which consist of 724,814 males. Of these individuals, a total of 16,359 developed UTI, with 28.9% having lower UTI and 71.1% having upper UTI.

### Study design

This study was approved by the ethics committee of our institute. Given the anonymous nature of the data, informed consent was not required, and the study received a waiver for informed consent. This study complies with the Declaration of Helsinki. The study period for the original cohort was between 2002 and 2018, and 4 years of washout (2002–2006) and 15 years of follow-up (2007–2018) were chosen. Patients newly diagnosed with PC were identified as those with KCD-8 code C61.

**Fig. 1:**
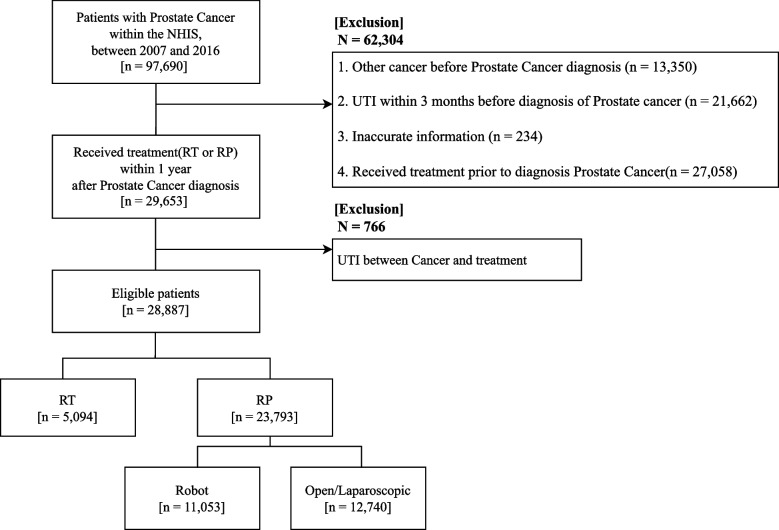
Flow chart of the study design. PC, prostate cancer; NHIS, National Health Insurance Service; UTI, urinary tract infection; RP, radical prostatectomy; RT, radiation therapy

A total of 97,690 patients were diagnosed with PC between 2007 and 2016. Patients who were diagnosed with any other cancer before PC (*n* = 13,350), who were diagnosed with UTIs within 3 months before PC diagnosis (*n* = 21,662), who had inaccurate information (*n* = 234), and who were treated with RT or RP prior to PC diagnosis (*n* = 27,058) were excluded. Patients with PC with a record of RT or RP treatment within a year after diagnosis were included (*n* = 29,653). Patients who developed UTIs between the date of PC diagnosis and the date of treatment with RT or RP were also excluded (*n* = 766). Thus, a total of 28,887 patients were eligible for our study (Fig. 1).

### Outcomes and variables

The primary outcome was UTI occurrence identified using KCD codes. UTI subtypes, namely, upper and lower UTI, were also considered. For subgroup analysis, the mortality of patients with UTIs was considered.

Primary treatment was defined as the initial treatment with RT, open/laparoscopic RP, and robot-assisted RP. Open/laparoscopic RP was identified using surgical codes. As robot-assisted RP is generally not covered by the NHIS, we defined robot-assisted RP as a record of pathological diagnosis and anesthesia after PC diagnosis without surgical codes. RT was identified using treatment codes. Disease and treatment codes are provided in the Supplementary Material (Table S1 and S2).

The age at PC diagnosis, year of PC diagnosis, ADT usage (within 3 months before or after primary treatment), and anticholinergic drug usage were assessed. Comorbidities included diabetes, renal disease, and hypertension. Charlson Comorbidity Index (CCI) scores, which is a measure of comorbidities, and known as a risk factor of prognostic of PC and overall survival, were included and categorized into three groups: 0–1, 2–3, and ≥ 4 [14, 15]. The latency period from PC to UTI (< 3 months, 3–6 months, 6–12 months, and > 12 months), UTI subtype (upper or lower), hospitalization, and radiation cystitis were also examined.

### Statistical analyses

Demographic and clinical characteristics were expressed as means ± standard deviations or numbers with percentages. Differences between the groups were compared using Student’s t-test and ANOVA for continuous variables and chi-squared test for categorical variables. Turkey correction was used for multiple comparisons. The standardized incidence ratio (SIR) with a 95% confidence interval (CI) was calculated to compare the UTI incidence rate of patients with PC with that of the general population. The UTI incidence rate of the general population was obtained from the HIRA-NPS. Kaplan–Meier curves were generated to compare the differences in UTI incidence and overall survival between treatment groups, age group at PC diagnosis, and year of PC diagnosis. Univariable and multivariable Cox proportional hazard models were used to estimate the hazard ratios (HRs) to examine the association between risk factors and the occurrence of UTIs and mortality for PC patients. The proportional hazard assumption test was performed using the scaled Schoenfeld residuals based on a multivariable Cox proportional hazard model [16]. Because the year of PC diagnosis and treatments violated the proportional hazard assumption, the Cox regression models were stratified by the year of PC diagnosis and included the time-varying effects of treatments that changed at 20 months of follow-up. Follow-up started on the date of the primary treatment. For the primary outcome, the follow-up ended on the date of UTI diagnosis, the date of death, or December 31, 2018, whichever came first. For the secondary outcome of mortality for PC patients with UTIs, the follow-up ended on the date of death or December 31, 2018, whichever came first. Subgroup analyses were performed for each outcome according to age group at PC diagnosis, and CCI. To evaluate the effect modification by age group at PC diagnosis and CCI for associations of treatments with each outcome, interactions between age group at PC diagnosis and treatments were included in the multivariable Cox models and significance tests for those interaction effects were performed.

Data were statistically analyzed using SAS v.7.0 (SAS Institute Inc., Cary, NC, USA) and R software, version 4.0.1 (R Foundation for Statistical Computing, Vienna, Austria). Data with *p* < 0.05 were considered statistically significant.

## Results

### Baseline characteristics of patients

A total of 28,887 patients with PC were included in this study: 5,094 (17.6%) with RT, 12,740 (44.1%) with open/laparoscopic RP, and 11,053 (38.3%) with robot-assisted RP. The patients’ mean age was 67.4 years at diagnosis. Their baseline characteristics are summarized in Table 1. Patients treated with RT (70.4 years) were older than those treated with open/laparoscopic RP (67.2 years) and robot-assisted RP (66.3 years). The year of PC diagnosis significantly differed between the treatment groups (*p* < 0.001). The use of ADT was higher in the RT group than in the RP groups (*p* < 0.001). The number of patients with CCI scores of 2 and 3 was higher in the RP groups than that in the RT group (*p* = 0.023 for RT vs. open/laparoscopic RP and *p* < 0.001 for RT vs. robot-assisted RP).

**Table 1:** Baseline characteristics of study population according to treatments

	Total	RT	RP Open/laparoscopic	RP Robot-assisted	*p*-value			
					ANOVA^a^	Pairwise^b^		
Characteristics	(*N* = 28,887)	(*N* = 5,094)	(*N* = 12,740)	(*N* = 11,053)		RT vs. Open/laparoscopic	RT vs. Robot-assisted	Robot-assisted vs. Open/laparoscopic
Age at PC diagnosis (years), mean (SD)	67.4 (7.3)	70.35 (7.9)	67.22 (6.1)	66.26 (7.9)	< 0.001	< 0.001	< 0.001	< 0.001
Age Group at PC diagnosis					< 0.001	< 0.001	< 0.001	< 0.001
- < 75	24,363 (84.3)	3,377 (66.3)	11,471 (90.0)	9,515 (86.1)				
- ≥ 75	4,524 (15.7)	1,717 (33.7)	1,269 (10.0)	1,538 (13.9)				
Year of PC diagnosis					< 0.001	< 0.001	< 0.001	< 0.001
- 2007–2009	6,516 (22.6)	1,323 (26.0)	3,579 (28.1)	1,614 (14.6)	< 0.001	0.006	< 0.001	< 0.001
- 2010–2012	9,798 (33.9)	1,614 (31.7)	4,457 (35.0)	3,727 (33.7)	< 0.001	< 0.001	0.299	0.099
- 2013–2016	12,573 (43.5)	2,157 (42.3)	4,704 (36.9)	5,712 (51.7)	< 0.001	< 0.001	< 0.001	< 0.001
UTI					< 0.001	< 0.001	0.226	< 0.001
- NO	22,569 (78.1)	4,164 (81.7)	9,497 (74.5)	8,908 (80.6)				
- YES	6,318 (21.9)	930 (18.3)	3,243 (25.5)	2,145 (19.4)				
ADT					< 0.001	< 0.001	< 0.001	< 0.001
- NO	21,806 (75.5)	1,876 (36.8)	10,919 (85.7)	9,011 (81.5)				
- YES	7,081 (24.5)	3,218 (63.2)	1,821 (14.3)	2,042 (18.5)				
Anticholinergic drugs					< 0.001	< 0.001	< 0.001	< 0.001
- NO	17,759 (61.5)	4,297 (84.4)	6,897 (35.8)	6,565 (44.4)				
- YES	11,128 (38.5)	797 (15.6)	5,843 (64.2)	4,488 (55.6)				
CCI scores					0.001	0.909	0.735	0.898
- 0–1	6,718 (23.3)	1,255 (24.6)	2,972 (23.3)	2,491 (22.5)	0.013	0.148	0.009	0.320
- 2–3	10,089 (34.9)	1,670 (32.8)	4,441 (34.9)	3,978 (36.0)	< 0.001	0.023	< 0.001	0.161
- ≥ 4	12,080 (41.8)	2,169 (42.6)	5,327 (41.8)	4,584 (41.5)	0.416	0.617	0.381	0.856
Diabetes					< 0.001	0.039	0.181	< 0.001
- NO	18,307 (63.4)	3,244 (63.7)	7,865 (61.7)	7,198 (65.1)				
- YES	10,580 (36.6)	1,850 (36.3)	4,875 (38.3)	3,855 (34.9)				
Renal disease					< 0.001	0.002	< 0.001	0.005
- NO	27,270 (94.4)	4,737 (93.0)	12,010 (94.3)	10,523 (95.2)				
- YES	1,617 (5.6)	357 (7.0)	730 (5.7)	530 (4.8)				
Hypertension					< 0.001	< 0.001	< 0.001	< 0.001
- NO	23,522 (81.4)	4,003 (78.6)	10,334 (81.1)	9,185 (83.1)				
- YES	5,365 (18.6)	1,091 (21.4)	2,406 (18.9)	1,868 (16.9)				

Values are presented as numbers (%) or means (standard deviations)

*Abbreviations*: *PC* Prostate cancer, *SD* Standard deviation, *UTI* Urinary tract infection, *ADT* Androgen deprivation therapy, *CCI* Charlson Comorbidity Index

^a^ANOVA

^b^Pairwise t-test with Tukey correction

**Fig. 2:**
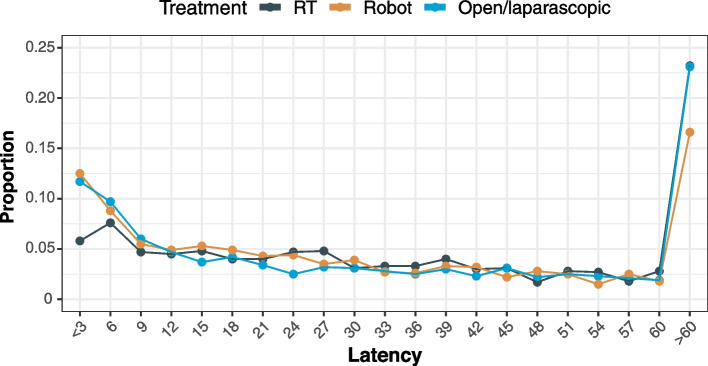
Line graph showing the proportion of UTI incidence for each treatment by latency

The characteristics of patients with UTIs are presented in Table 2. The RP groups had a higher incidence of UTIs within 3 months after PC diagnosis than the RT group (*p* < 0.001 for RT vs. open/laparoscopic RP and *p* < 0.001 for RT vs. robot-assisted RP). However, the RT group had a higher incidence of UTIs 12 months after PC diagnosis than the RP groups (*p* < 0.001 for RT vs. open/laparoscopic RP and *p* < 0.001 for RT vs. robot-assisted RP). Of the 6,318 patients with UTIs, 1,064 (16.8%) and 5,254 (83.2%) had upper and lower UTIs, respectively. SIRs were 2.47 (95% CI, 2.35–2.60; *p* < 0.001) for upper UTIs and 3.04 (95% CI, 2.95–3.12; *p* < 0.001) for lower UTIs. The proportion of UTI incidence decreased dramatically in RP groups and slowly in RT group after the curative treatments (Fig. 2).

**Table 2:** Baseline characteristics of patients with UTIs according to treatments

	Total	RT	RP Open/laparoscopic	RP Robot-assisted	*p*-value			
					ANOVA^a^	Pairwise^b^		
Characteristics	(*N* = 6,318)	(*N* = 930)	(*N* = 3,243)	(*N* = 2,145)		RT vs. Open/laparoscopic	RT vs. Robot-assisted	Robot-assisted vs. Open/laparoscopic
Age at diagnosis of UTI, mean (SD)	70.69 (7.2)	73.76 (7.5)	70.72 (6.5)	69.89 (7.8)	< 0.001	< 0.001	< 0.001	< 0.001
Age Group					< 0.001	< 0.001	< 0.001	< 0.001
- < 75	5,309 (84.0)	622 (66.9)	2,883 (88.9)	1,804 (84.1)				
- ≥ 75	1,009 (16.0)	308 (33.1)	360 (11.1)	341 (15.9)				
Latency from PC to UTI (months), mean (SD)	32.76 (30.9)	38.76 (30.7)	36.51 (32.8)	31.34 (27.6)	< 0.001	0.120	< 0.001	< 0.001
Latency from PC to UTI (months)					< 0.001	< 0.001	< 0.001	0.996
- < 3	730 (11.1)	54 (5.8)	380 (11.7)	269 (12.5)	< 0.001	< 0.001	< 0.001	0.613
- 3–6	575 (9.1)	71 (7.6)	316 (9.7)	188 (8.8)	0.115	0.119	0.576	0.439
- 6–12	660 (10.4)	86 (9.2)	349 (10.8)	241 (11.2)	0.411	0.378	0.555	0.945
- > 12	4,380 (69.3)	719 (77.3)	2,198 (67.8)	1,420 (66.2)	< 0.001	< 0.001	< 0.001	0.940
UTI type					0.069	0.444	0.067	0.282
- UPPER	1,561 (24.7)	251 (27.0)	812 (25.0)	498 (23.2)				
- LOWER	4,757 (75.3)	679 (73.0)	2,431 (75.0)	1,647 (76.8)				
Hospitalization at UTI diagnosis					0.021	0.297	0.871	0.019
- NO	5,574 (88.2)	827 (88.9)	2,826 (87.1)	1,921 (89.6)				
- YES	744 (11.8)	103 (11.1)	417(12.9)	224 (10.4)				
Radiation cystitis					< 0.001	< 0.001	< 0.001	0.323
- NO	6,239 (98.7)	898 (96.6)	3,209 (99.0)	2,132 (99.4)				
- YES	79(1.3)	32 (3.4)	34 (1.0)	13 (0.6)				

Values are presented as numbers (%) or means (standard deviations)

*Abbreviations*: *PC* prostate cancer, *SD* standard deviation, *UTI* urinary tract infection, *ADT* androgen deprivation therapy, *CCI* Charlson Comorbidity Index

^a^ANOVA

^b^Pairwise t-test with Tukey correction

### Occurrence risk of UTIs in patients and mortality risk in patients with UTIs

**Fig. 3:**
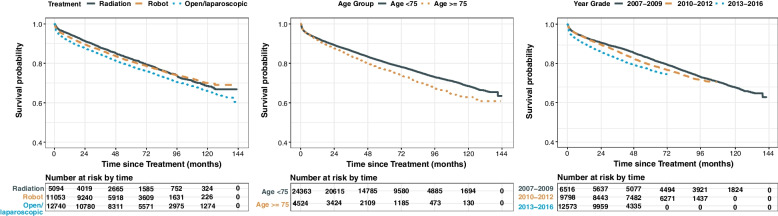
Kaplan–Meier survival curves of urinary tract infection according to treatments, age group at PC diagnosis, and year of PC diagnosis. UTI, urinary tract infection; RT, radiation therapy

Figure 3 displays the Kaplan–Meier survival curves of UTI incidence stratified by treatment, age at PC diagnosis, and year of PC diagnosis. The RT group had higher survival probabilities for UTI incidence than the RP groups during the early follow-up, and after 6 years of follow-up, the robot-assisted RP group had the highest survival probabilities for UTI incidence. Survival probabilities for UTI incidence for younger age and an early year of PC diagnosis were higher. The Kaplan-Meir survival curves of mortality for patients with UTIs show that the RP group, younger age, and a later year of PC diagnosis were associated with higher survival probabilities (Fig. S1). Table 3 presents the unadjusted HRs and fully adjusted HRs (aHRs) for UTI incidence. In the early follow-up period, the risk of UTIs was higher in the open/laparoscopic RP group (aHR, 1.63; 95% CI, 1.44–1.83; *p* < 0.001) and the robot-assisted RP group (aHR, 1.26; 95% CI, 1.11–1.43; *p* < 0.001), compared to the RT group. However, in the late follow-up period, there was no significant difference in the risk of UTIs between the RT group and both the open/laparoscopic RP group (aHR, 1.02; 95% CI, 0.78–1.34; *p* = 0.887) and the robot-assisted RP group (aHR, 0.92; 95% CI, 0.70–1.23; *p* = 0.573). The robot-assisted RP group had a lower risk of UTIs than the open/laparoscopic RP group in the early (aHR, 0.77; 95% CI, 0.77–0.78; *p* < 0.001) and late (aHR, 0.90; 95% CI, 0.89–0.91; *p* < 0.001) follow-up periods. Patients who were diagnosed of PC at an older age (aHR, 1.19; 95% CI, 1.11–1.28; *p* < 0.001), had diabetes (aHR, 1.10; 95% CI, 1.04–1.16; *p* = 0.001), had renal disease (aHR, 1.30; 95% CI, 1.19–1.43; *p* < 0.001), received ADT (aHR, 1.13; 95% CI, 1.06–1.20; *p* < 0.001), and used anticholinergic drugs (aHR, 1.34; 95% CI, 1.27–1.41; *p* < 0.001) had a higher risk of UTIs. Table 4 shows the HRs and aHRs of the overall survival of patients with UTIs. In the early follow-up period, the risk of death for patients with UTIs was lower in both the open/laparoscopic RP (aHR, 0.43; 95% CI, 0.33–0.55; *p* < 0.001) and the robot-assisted RP (aHR, 0.68; 95% CI, 0.53–0.88; *p* = 0.004) groups than in the RP group. However, the difference became insignificant thereafter. Patients diagnosed with upper UTIs had a higher mortality rate than those diagnosed with lower UTIs (aHR, 1.23; 95% CI, 1.05–1.44; *p* = 0.010). Patients who had UTIs and hospitalized at diagnosis had a higher mortality rate than those who were not hospitalized (aHR, 1.91; 95% CI, 1.59–2.30; *p* < 0.001). Sepsis from UTIs increased the mortality rate of patients (aHR, 2.63; 95% CI, 1.39–4.96; *p* = 0.003). On subgroup analysis, there were no significant differences in the association of treatments with the risk of UTIs by age group of PC diagnosis and CCI, however, significant differences were observed in the association of treatments with mortality (Table S3).

**Table 3:** Univariable and multivariable cox regression analyses of UTIs in the study population

Variables	Number of patients at risk	No. (%)	Person-years	Incidence rate (per 1,000 person-years)	Univariable		Multivariable	
					HR (95% CI)	*p*-values	HR (95% CI)	*p*-values
Treatment								
- Radiation	5,094	930 (18.3)	23546.5	39.5	1 (reference)		1 (reference)	
- $<$ 20 m Robot-assisted	11,053	1,021 (9.2)	17043.2	59.9	1.27 (1.13 – 1.44)	< 0.001	1.26 (1.11 – 1.43)	< 0.001
- $\ge$ 20 m Robot-assisted	9,666	1,124 (11.6)	34597.2	32.5	0.91 (0.69 – 1.20)	0.504	0.92 (0.70 – 1.23)	0.573
- $<$ 20 m Open/laparoscopic	12,740	1,439 (11.3)	19431.0	74.1	1.57 (1.40 – 1.76)	< 0.001	1.63 (1.44–1.83)	< 0.001
- $\ge$ 20 m Open/laparoscopic	11,091	1,804 (16.3)	50555.4	35.7	1.01 (0.78 – 1.31)	0.940	1.02 (0.78 – 1.34)	0.887
Age Group at PC diagnosis								
- $<$ 75 y	24,363	5,309 (21.8)	126339.1	42.0	1 (reference)		1 (reference)	
- $\ge$ 75 y	4,524	1,009 (22.3)	18847.1	53.5	1.22 (1.14 – 1.30)	< 0.001	1.19 (1.11 – 1.28)	< 0.001
CCI score								
- 0–1	6,718	1,201 (17.9)	38550.1	31.2	1 (reference)		1 (reference)	
- 2–3	10,089	2,082 (20.6)	52526.6	39.6	1.25 (1.17–1.34)	< 0.001	1.20 (1.12–1.29)	< 0.001
- ≥ 4	12,080	3,035 (25.1)	54109.4	56.1	1.72 (1.61–1.84)	< 0.001	1.53 (1.42–1.64)	< 0.001
Diabetes								
- NO	18,307	3,666 (20.0)	92335.5	39.7	1 (reference)		1 (reference)	
- YES	10,580	2,652 (25.1)	52850.6	50.2	1.26 (1.20–1.33)	< 0.001	1.10 (1.04–1.16)	0.001
Renal disease								
- NO	27,270	5,826 (21.4)	137236.8	42.5	1 (reference)		1 (reference)	
- YES	1,617	492 (30.4)	7949.4	61.9	1.45 (1.32–1.59)	< 0.001	1.30 (1.19–1.43)	< 0.001
Hypertension								
- NO	23,522	4,975 (21.2)	117097.2	42.5	1 (reference)		1 (reference)	
- YES	5,365	1,343 (25.0)	28089.0	47.8	1.14 (1.07–1.21)	< 0.001	1.01 (0.95–1.08)	0.707
ADT								
- NO	21,806	4,847 (22.2)	113275.4	42.8	1 (reference)		1 (reference)	
- YES	7,081	1,471 (20.8)	31910.8	46.1	1.05 (0.99–1.11)	0.134	1.13 (1.06–1.20)	< 0.001
Anticholinergic drugs								
- NO	17,759	3,489 (19.7)	92257.5	37.8	1 (reference)		1 (reference)	
- YES	11,128	2,829 (25.4)	52928.7	53.4	1.39 (1.32–1.46)	< 0.001	1.34 (1.27–1.41)	< 0.001

The model was adjusted for age at PC diagnosis, period of PC diagnosis, CCI score, diabetes, renal disease, hypertension, anticholinergic drug, and androgen deprivation therapy

*Abbreviations*: *OS* overall survival, *PC* prostate cancer, *UTI* urinary tract infection, *ADT* androgen deprivation therapy, *CCI* Charlson Comorbidity Index

**Table 4:** Univariable and multivariable Cox regression analyses of the OS of patients with UTIs

Variables	Number of patients at risk	No. (%)	Person-years	Incidence rate (per 1,000 person-years)	Univariable		Multivariable	
					HR (95% CI)	*p*-values	HR (95% CI)	*p*-values
Treatment								
- Radiation	930	237 (25.5)	5875.6	40.3	1 (reference)		1 (reference)	
- $<$ 20 m Robot-assisted	2,145	130 (6.1)	3115.6	41.7	0.48 (0.38 – 0.62)	< 0.001	0.68 (0.53 – 0.88)	0.004
- $\ge$ 20 m Robot-assisted	1,597	123 (7.7)	4399.0	28.0	0.41 (0.23 – 0.77)	0.006	0.59 (0.32 – 1.09)	0.092
- $<$ 20 m Open/laparoscopic	3,243	136 (4.2)	4866.2	27.9	0.33 (0.25 – 0.42)	< 0.001	0.43 (0.33 – 0.55)	< 0.001
- $\ge$ 20 m Open/laparoscopic	2,584	249 (9.6)	8517.9	29.2	0.43 (0.24 – 0.76)	0.004	0.57 (0.32—1.02)	0.058
Age Group at PC diagnosis								
- $<$ 75 y	5,309	606 (11.4)	35549.8	17.0	1 (reference)		1 (reference)	
- $\ge$ 75 y	1,009	269 (26.7)	5818.3	46.2	2.73 (2.36 – 3.15)	< 0.001	1.05 (0.86 – 1.29)	0.635
CCI score								
- 0–1	1,201	198 (16.5)	8645.6	22.9	1 (reference)		1 (reference)	
- 2–3	2,082	248 (11.9)	14061.8	17.6	0.73 (0.61 – 0.88)	0.001	0.75 (0.62 – 0.91)	0.003
- ≥ 4	3,035	429 (14.1)	18660.7	23.0	0.95 (0.81 – 1.13)	0.584	0.90 (0.75 – 1.09)	0.283
Diabetes								
- NO	3,666	475 (13.0)	23589.1	20.1	1 (reference)		1 (reference)	
- YES	2,652	400 (15.1)	17779.0	22.5	1.14 (1.00 – 1.30)	0.050	1.045 (0.90 – 1.20)	0.560
Renal disease								
- NO	5,826	779 (13.4)	37978.1	20.5	1 (reference)		1 (reference)	
- YES	492	96 (19.5)	3390.0	28.3	1.45 (1.17 – 1.79)	< 0.001	1.16 (0.93 – 1.44)	0.187
Age at UTI diagnosis	6,318	875 (13.9)	41368.1	21.2	1.10 (1.09 – 1.11)	< 0.001	1.08 (1.07 – 1.10)	< 0.001
UTI within 3 months after treatment								
- NO	5,054	731 (14.5)	35126.9	20.8	1 (reference)		1 (reference)	
- YES	1,264	144 (11.4)	6241.2	23.1	0.57 (0.48 – 0.69)	< 0.001	0.78 (0.64 – 0.94)	0.011
UTI type								
- LOWER	5,254	603 (11.5)	31022.0	19.4	1 (reference)		1 (reference)	
- UPPER	1,064	272 (25.6)	10346.1	26.3	1.73 (1.50 – 2.00)	< 0.001	1.23 (1.05 – 1.44)	0.010
Hospitalization at UTI diagnosis								
- NO	5,574	704 (12.6)	36617.9	19.2	1 (reference)		1 (reference)	
- YES	744	171 (23.0)	4750.2	36.0	2.29 (1.93 – 2.70)	< 0.001	1.91 (1.59 – 2.30)	< 0.001
Sepsis from UTI								
- NO	6,289	865 (13.8)	41232.8	21.0	1 (reference)		1 (reference)	
- YES	29	10 (34.5)	135.3	73.9	2.8 (1.50 – 5.23)	< 0.001	2.63 (1.39 – 4.96)	0.003

The model was adjusted for age at PC diagnosis, period of PC diagnosis, CCI score, diabetes, renal disease, age at UTI diagnosis, UTI within 3 months after treatment, UTI type, hospitalization, and sepsis from UTI

*Abbreviations*: *OS* Overall survival, *PC* Prostate Cancer, *UTI* urinary tract infection, *ADT* Androgen deprivation therapy, *CCI* Charlson Comorbidity Index

## Discussion

In this nationwide population-based study, the incidence of UTIs in RT- or RP-treated patients with PC was much higher than that in the general population. Patients treated with RP had a higher risk of UTIs than those treated with RT in the early follow-up period; however, the robot-assisted RP group had a lower risk of UTIs than the open/laparoscopic RP group in total follow-up period. After the primary treatment, the age at UTI diagnosis, upper UTIs, hospitalization, and sepsis from UTIs were factors related to the poor overall survival of patients with UTIs.

The incidence of UTIs after RT or RP remains relatively unclear. In a prospective study involving approximately 425 robot-assisted RP-treated patients, the UTI incidence rate was 6.1% 30 days after surgery [17]. In another study, external beam RT with a transponder caused infectious complications in 10% of the participants in several weeks [18]. However, the reported incidence can be affected by follow-up duration, treatment procedure, or survey methods [19]. In our nationwide cohort, UTIs occurred in 21.9% of the patients at a median of 5.76 years of follow-up. Some factors might cause the increased incidence rate of UTIs after PC treatment compared with that in the general population. First, surveillance was conducted densely and regularly to detect cancer recurrence in patients with PC. A timely follow-up schedule is important to prevent delays in detecting the progression and worsening of complications [20]. Second, treatment-induced anatomical or histological changes can increase UTI occurrence. UTI-causing bacteria can ascend through the urethra into the bladder, and RP can weaken the defense mechanism of males with a long urethra [21]. RT can induce submucosal and mucosal changes by destroying small arteries or causing fibrosis [22]. Vascular injury can induce ischemic changes and destroy mucosal continuity [22]. RT-induced fibrosis caused by inflammation can occur after months or years [23]. Therefore, RT-induced chronic histological changes can be associated with different tendencies of UTI occurrence, i.e., higher in the acute phase of RP and the chronic phase of RT. We hypothesized that radiation cystitis would affect the difference in UTI tendencies between the RT and RP groups. Although the incidence of radiation cystitis significantly differed between the groups, it accounted for a small portion of the total UTI cases.

UTI incidence rates differed among various RP methods. Although robot-assisted RP shows similar functional outcomes to open RP in one RCT [24], its functional outcomes are superior to open/laparoscopic RP in several community-based studies [25]. With the rapid development of minimally invasive surgery with small surgical incisions, open RP has been replaced by laparoscopic RP. Eventually, robot-assisted RP has become the preferred option. In the recent period (2013–2016) of our study, robot-assisted RP accounted for more than half of all RPs. Although robot-assisted RP is costly, it is preferred by surgeons to laparoscopic RP because of its shorter learning curve and easier instrument handling [26]. Robot-assisted RP is also associated with fewer postoperative complications and a better urinary continence rate than laparoscopic RP [25]. These advantages of robot-assisted RP may result in a reduced possibility of UTIs. Postoperative UTIs can be associated with bladder microbiome composition, which is changed by incontinence surgery [27]. In our study, patients who used anticholinergic drugs before the primary treatment were fragile at UTI. The possible pathophysiology of overactive bladder syndrome includes increased voiding pressure, impaired barrier function, and increased urothelial apoptosis [28].

We reported differences in UTI occurrence according to different treatments for patients with PC, but we did not report increased UTI risks because this study used a retrospective design, and selection bias was not completely corrected despite an adjusted multivariable model. The survival of patients in the RP group was significantly higher than that in the RT. In an almost nationwide population-based observational study, RP results in better survival rates than RT does; however, in an RCT, RP and RT yield similar survival rates [6, 29]. This difference might be attributed to confounding factors that could not be controlled in a nationwide dataset. However, our findings suggested that the characteristics of patients with UTIs could be associated with their prognosis. In the elderly population, underlying comorbidities are common risk factors for UTIs. In our study, relatively healthy patients with UTIs and low CCI scores had worse survival than those with high CCI scores. These paradoxical results might be attributed to impaired immune mechanisms against UTI-causing pathogens. The immune system is an important defense mechanism against exogenous agents, including cancer cells [30]. Primary RT could trigger immune changes by suppressing anticancer immunity [31]. ADT can remodel the tumor immune microenvironment [32]. With changes in immunity during PC treatment, the severity of UTIs (including upper UTIs), hospitalization, or sepsis could indicate patients’ vulnerability to PC.

Our study has several limitations. First, the NHIS database does not provide the tumor stage. We included definitive therapies, namely, RT and RP; therefore, we hypothesized that metastatic disease would be excluded. The comparison of the incidence of UTIs between curative and palliative treatments could be an interesting future research topic. Second, abnormal imaging findings, such as hydronephrosis and trabeculation, could not be evaluated. Abnormal anatomical defects could affect the risk of UTIs. Lastly, the exact NHIS records of robot-assisted RP could not be found because it is a noncovered service. Therefore, we used concomitant anesthetic and pathological codes in the absence of surgical codes, which were used in another study to minimize the misclassification of robot-assisted RP [33]. In addition, this study may be a complete enumeration of prostate cancer survey in South Korea because expensive cancer treatments and examinations have become more affordable since being covered by the NHIS. However, it is possible that the diagnosis of UTI was missed because relatively low cost of treatment of UTI may have discouraged some patients from visiting a hospital, as a result, the diagnosis of UTI was not recorded by a physician.

## Conclusion

In RP- or RT-treated patients, the incidence of UTI was higher than that in the general population. The period of UTI occurrence differed between the RP and RT groups. RP posed a higher risk of UTIs than RT did in early follow-up period. Robot-assisted RP had a lower risk of UTIs than open/laparoscopic RP group in total period. The severity characteristics of UTIs might be related to poor prognosis.

## Supplementary Information

**Additional file 1:** **Table S1.** KCD-8codesof diagnoses used for defining the study population, comorbidities, andoutcomes. **Table S2.** The HealthInsurance Review and Assessment (HIRA) codes of treatments used in the study. **Table S3.** Multivariable Cox Regression Analysesof the UTIs and the OS According to Age Group and CCI. **Figure S1.** Kaplan-Meirsurvival curves of mortality for patients with UTIs according to treatments,age group at diagnosis, and year of diagnosis.

## Data Availability

The data that support the findings of this study are available from the National Health Insurance Service but restrictions apply to the availability of these data, which were used under license for the current study, and so are not publicly available. Data are however available from the authors upon reasonable request and with permission of the National Health Insurance Service. Analysed data can be contacted through direct contact with the author, Jihye Hyun (zeze111111@cau.ac.kr).
