# Supplementary material for: Urinary tract infection after radiation therapy or radical prostatectomy on the prognosis of patients with prostate cancer: a population-based study
Source: BMC Cancer. 2023 May 3;23:395. doi: 10.1186/s12885-023-10869-4 (PMC10157974; doi:10.1186/s12885-023-10869-4)

Table S1. KCD-8codes of diagnoses used for defining the study population, comorbidities, and outcomes

| Diagnosis | KCD-8 codes^¶^ |
| --- | --- |
| **Study population selection** |  |
| Prostate cancer | C61 |
| Any type of cancer | C00-C97 (except for C61) |
| **Outcome** |  |
| UTI | Upper UTI : N10, N110, N111, N118, N119, N12, N151 / Lower UTI : N300, N302, N308, N309 / N304 |
| **Comorbidity condition** |  |
| Diabetes | E100, E105-E108, E110, E115-E118, E120-E130, E135-E138, E140, E145-E148 |
| Hypertension | I10-I13, I15 |
| Moderate to severe renal disease | I120, I131, N302, N033-N037, N052-N057, N19, N250, Z490-Z492, Z940, Z992 |
| **Charlson comorbidity index** |  |
| Hypertension | I10-I13, I15 |
| Diabetes | E100, E105-E108, E110, E115-E118, E120-E130, E135-E138, E140, E145-E148 |
| Myocardial infarction | I21, I22 |
| Congestive heart failure | I099, I110, I130, I132, I255, 1420, 1425-I429, I43, P290 |
| Peripheral vascular disease | I731, I738, I771, I792, K551, K558, K559, I70 |
| Cerebrovascular disease | G12, G46, H340, I61-I68 |
| Dementia | F03, G30, F051, G311 |
| Chronic lung disease | J40, J67, I278, I279, J684, J701, J703 |
| Rheumatic disease | M060-M064, M068, M069, M315, M330, M331, M339, M351, M360 |
| Peptic ulcer | K250-K253, K259, K260-K263, K269-K273, K279-K283, K289 |
| Mild liver disease | K700-K702, K709, K713-K715, K740-K742, K760, K762-K764, K768, K769, Z944, B18 |
| Diabetes mellitus without end-organ | E100, E106, E108, E110, E116, E118, E120, E121, E126, E128-E130, E136, E138, E140, E146, E14 |
| Diabetes mellitus with end-organ | E105, E107, E115, E117, E122-E125, E127, E135, E137, E145, E147 |
| Hemiplegia | G041, G114, G801, G802, G823-G825, G830-G834, G839 |
| Moderate to severe renal disease | I120, I131, N302, N033-N037, N052-N057, N19, N250, Z490-Z492, Z940, Z992 |
| Moderate to severe liver disease | I850, I859, I864, I982, K704, K711, K765 |
| AIDS | B24 |

^¶^ Korean Standard Classification of Diseases, 8th revision by Statistics Korea

Abbreviations: UTI, urinary tract infection; AIDS, acquired immune deficiency syndrome

Table S2. The Health Insurance Review and Assessment (HIRA) codes of treatments used in the study

|  | HIRA codes^§^ |
| --- | --- |
| **Treatments** |  |
| Open/laparoscopic | R3950, R3960 |
| Prostate biopsy | C8551, C8552 |
| Tissue pathology | C5916, C5917, C5500, C5504, C5918, C5919, C5508, C5505 |
| Anaesthesia | L0101, L1211, L1221, L1212, L1222, LX001, L1330, L1310, L1320 |
| Radiation | HD010-HD020, HD031-HD033, HD041, HD051-HD059, HD061, HD071-HD073, HD110-HD115, HD121, HD211-HD212, HD410-HD420, HD441, HH121-123, HH131-HD140, HZ271-HZ274, HZ331-HZ336 |

^§^ The Health Insurance Review and Assessment (HIRA) assigns unified standard code to each treatment.

Table S3. Multivariable Cox Regression Analyses of the UTIs and the OS According to Age Group and CCI.

|  |  | UTI | | |  | Overall Survival |  | |  |
| --- | --- | --- | --- | --- | --- | --- | --- | --- | --- |
|  |  | HR (95% CI) | p-values | p-value for interaction | HR (95% CI) | p-values | p-value for interaction | |  |
| Age Group at PC diagnosis |  |  |  | 0.055 |  |  | 0.002 | |  |
| Age at PC diagnosis $<$75 |  |  |  |  |  |  |  | |  |
| - Radiation |  | 1 (reference) |  |  | 1 (reference) |  | |  | |
| - $<$20 m Robot-assisted |  | 1.14 (0.98 – 1.32) | 0.091 |  | 0.48 (0.35 – 0.66) | <0.001 | |  | |
| - $\geq$20 m Robot-assisted |  | 0.92 (0.65 – 1.28) | 0.621 |  | 0.47 (0.21 – 1.02) | 0.056 | |  | |
| - $<$20 m Open/laparoscopic |  | 1.52 (1.32 – 1.76) | <0.001 |  | 0.32 (0.23 – 0.43) | <0.001 | |  | |
| - $\geq$20 m Open/laparoscopic |  | 0.99 (0.73 – 1.38) | 0.998 |  | 0.55 (0.27 – 1.11) | 0.095 | |  | |
| Age at PC diagnosis $\geq$75 |  |  |  |  |  |  | |  | |
| - Radiation |  | 1 (reference) |  |  | 1 (reference) |  | |  | |
| - $<$20 m Robot-assisted |  | 1.62 (1.28 – 2.04) | <0.001 |  | 1.08 (0.71 – 1.64) | 0.718 | |  | |
| - $\geq$20 m Robot-assisted |  | 0.93 (0.54 – 1.61) | 0.795 |  | 0.86 (0.32 – 2.33) | 0.767 | |  | |
| - $<$20 m Open/laparoscopic |  | 1.66 (1.30 – 2.12) | <0.001 |  | 0.71 (0.45 – 1.14) | 0.156 | |  | |
| - $\geq$20 m Open/laparoscopic |  | 1.08 (0.62 – 1.88) | 0.786 |  | 0.58 (0.20 – 1.70) | 0.321 | |  | |
| CCI |  |  |  | 0.449 |  |  | | 0.048 | |
| CCI 0-1 |  |  |  |  |  |  | |  | |
| - Radiation |  | 1 (reference) |  |  | 1 (reference) |  | |  | |
| - $<$20 m Robot-assisted |  | 1.33 (0.98 – 1.79) | 0.064 |  | 0.67 (0.39 – 1.15) | 0.146 | |  | |
| - $\geq$20 m Robot-assisted |  | 0.91 (0.47 – 1.76) | 0.779 |  | 0.48 (0.13 – 1.77) | 0.270 | |  | |
| - $<$20 m Open/laparoscopic |  | 1.68 (1.26 – 2.24) | <0.001 |  | 0.44 (0.26 – 0.73) | 0.001 | |  | |
| - $\geq$20 m Open/laparoscopic |  | 0.90 (0.48 – 1.69) | 0.743 |  | 0.49 (0.15 – 1.61) | 0.240 | |  | |
| CCI 2-3 |  |  |  |  |  |  | |  | |
| - Radiation |  | 1 (reference) |  |  | 1 (reference) |  | |  | |
| - $<$20 m Robot-assisted |  | 1.39 (1.10 – 1.76) | 0.006 |  | 0.93 (0.57 – 1.52) | 0.772 | |  | |
| - $\geq$20 m Robot-assisted |  | 0.83 (0.49 – 1.40) | 0.485 |  | 0.57 (0.18 – 1.83) | 0.345 | |  | |
| - $<$20 m Open/laparoscopic |  | 1.86 (1.48 – 2.34) | <0.001 |  | 0.43 (0.25 – 0.72) | 0.001 | |  | |
| - $\geq$20 m Open/laparoscopic |  | 0.93 (0.56 – 1.54) | 0.778 |  | 0.53 (0.16 – 1.70) | 0.286 | |  | |
| CCI ≥4 |  |  |  |  |  |  | |  | |
| - Radiation |  | 1 (reference) |  |  | 1 (reference) |  | |  | |
| - $<$20 m Robot-assisted |  | 1.17 (0.99 – 1.39) | 0.070 |  | 0.57 (0.39 – 0.82) | 0.002 | |  | |
| - $\geq$20 m Robot-assisted |  | 1.02 (0.69 – 1.52) | 0.922 |  | 0.62 (0.26 – 1.49) | 0.285 | |  | |
| - $<$20 m Open/laparoscopic |  | 1.49 (1.26 – 1.75) | <0.001 |  | 0.43 (0.30 – 0.61) | <0.001 | |  | |
| - $\geq$20 m Open/laparoscopic |  | 1.16 (0.80 – 1.70) | 0.447 |  | 0.63 (0.28 – 1.44) | 0.273 | |  | |

Figure S1. Kaplan-Meir survival curves of mortality for patients with UTIs according to treatments, age group at diagnosis, and year of diagnosis


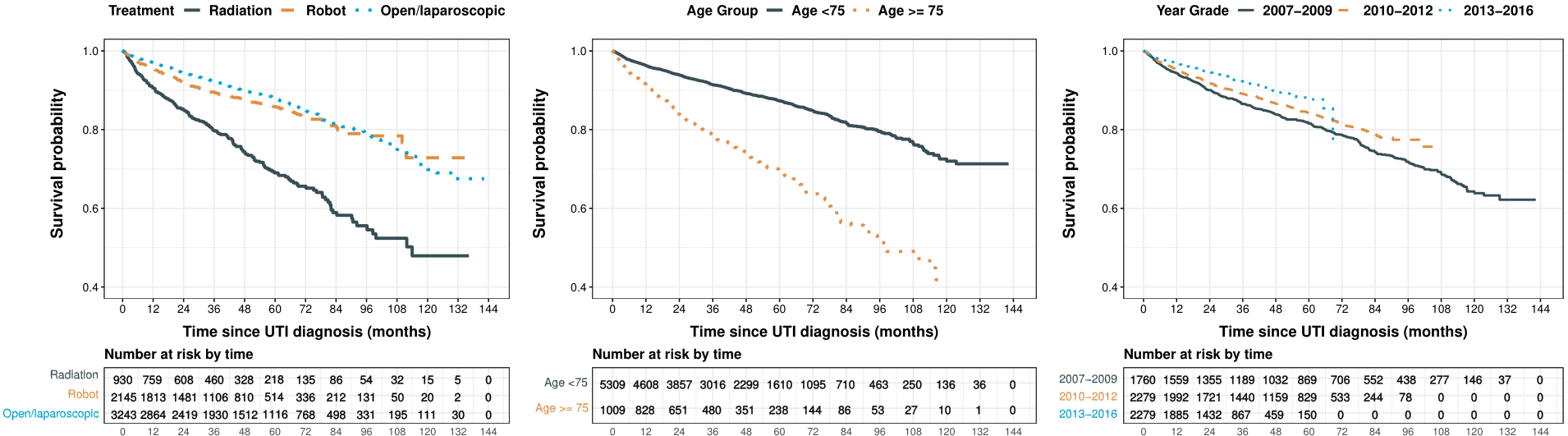

Supplement: Supplementary file 1 — Additional file 1: Table S1. KCD-8codesof diagnoses used for defining the study population, comorbidities, andoutcomes. Table S2. The HealthInsurance Review and Assessment (HIRA) codes of treatments used in the study. Table S3. Multivariable Cox Regression Analysesof the UTIs and the OS According to Age Group and CCI. Figure S1. Kaplan-Meirsurvival curves of mortality for patients with UTIs according to treatments,age group at diagnosis, and year of diagnosis. [file 12885_2023_10869_MOESM1_ESM.docx]
